# Innate Immune Sensing by Cells of the Adaptive Immune System

**DOI:** 10.3389/fimmu.2020.01081

**Published:** 2020-05-29

**Authors:** Tanja Stögerer, Simona Stäger

**Affiliations:** INRS Centre Armand-Frappier Santé Biotechnologie, Laval, QC, Canada

**Keywords:** TLRs, PRRs, DAMPs, B and T cells, adaptive immunity, chronic infections

## Abstract

Sensing of microbes or of danger signals has mainly been attributed to myeloid innate immune cells. However, T and B cells also express functional pattern recognition receptors (PRRs). In these cells, PRRs mediate signaling cascades that result in different functions depending on the cell's activation and/or differentiation status, on the environment, and on the ligand/agonist. Some of these functions are beneficial for the host; however, some are detrimental and are exploited by pathogens to establish persistent infections. In this review, we summarize the available literature on innate immune sensing by cells of the adaptive immune system and discuss possible implications for chronic infections.

## Introduction

For many years, sensing of conserved structures called pathogen-associated molecular patterns (PAMPs) or damage-associated molecular patterns (DAMPS) was thought to be a prerogative of myeloid cells of the innate immune system, such as macrophages, dendritic cells, or neutrophils. In the context of infectious diseases, innate immune sensing is responsible for launching a potent initial inflammatory response aimed to non-specifically eliminate invading pathogens. This initial defense mechanism is typically followed by a more specific and targeted response, which is orchestrated by cells of the adaptive immunity, namely B and T lymphocytes. It is owed to this notion that PAMP and DAMP sensing is extensively researched in innate immune cells, whereas innate immune sensing in lymphocytes has only recently been demonstrated and literature on physiological and pathological implications is still sparse. These mechanisms have perhaps been previously overlooked because lymphocyte activation typically occurs at later stages of infection, when inflammation is already established; however, recent evidence suggests that innate immune signaling can not only participate in lymphocyte maturation and improvement of B and T cell responses, but also be hijacked by pathogens such as *Leishmania donovani* to exacerbate detrimental immunosuppressive effects and induce hypergammaglobulinemia.

For the purpose of this short review, we will discuss the emerging field of innate immune sensing by cells of the adaptive immunity and its implications in *Leishmania* and other neglected tropical diseases such as Trypanosomiasis in two parts, placing individual focus on the two major actors of adaptive immunity, B and T lymphocytes.

## Innate Immune Sensing in b Cells

### Expression and Functions of Innate Immune Sensors in B Cells

Toll-Like Receptors (TLR) were the first innate immune sensors to be attributed a role in B cells. TLRs are a family lectin-rich repeats containing transmembrane proteins located on the cell surface (TLR1, TLR2, TLR4-6, and TLR10-11) or inside the endosome (TLR3, TLR7-9). Murine B cells express TLR1-4, TLR6, TLR7, and TLR9 at varying levels in different subsets (1), while TLR expression on human B cells includes TLR1, TLR2, TLR6, TLR7, TLR9, and TLR10 (2), and in the case of plasma cells also TLR3 and TLR4 (3). Several beneficial roles of TLR-mediated sensing in B cell have been demonstrated, including the promotion of B cell maturation through TLR4 stimulation (4, 5) and enhanced antigen presentation by TLR9 ligation (6). While TLR engagement has been proposed to act as an additional signal to B cell receptor (BCR) stimulation (7), B cells have been shown to produce various cytokines and chemokines solely from TLR triggering (8).

Recent discoveries of cytosolic innate immune sensing pathways, including sensing of cytosolic DNA involving the adaptor protein stimulator of interferon genes (STING, also termed MYPS, MITA, ERIS) or cytosolic RNA via the adaptor MAVS have greatly advanced our understanding of immunity. A variety of sensor proteins, such as cyclic GMP-AMP synthase (cGAS) and interferon-induced protein 16 (IFI16), have been proposed to directly interact with DNA, leading to enzymatic generation of a secondary messenger molecule in the form of cyclic dinucleotides (CDNs), such as 2′3′-cyclic guanosine monophosphate-adenosine monophosphate (cGAMP). These CDNs can then activate STING on surface of the endoplasmic reticulum to interact with TANK-binding kinase 1 (TBK1), resulting in phosphorylation of interferon regulatory factor 3 and subsequent IFN-I production (9).

Expression and functionality of STING and its pathway have also been demonstrated in B cells, although there is a discrepancy between cells of human and murine origin. Reports unanimously confirm STING expression in murine B cells, and have demonstrated that B cells are capable of responding to STING stimulation by production of IFN-I (10, 11); however, conflicting literature exists on STING expression and function in human B cells. In one study on peripheral blood mononuclear cell (PBMC)-derived human B cells, the presence of STING was confirmed via both flow cytometry and qPCR (12), while another study failed to detect STING via RT-qPCR in primary B cells from tonsils and PBMCs, but confirmed the expression of upstream (cGAS, IFI16) and downstream (TBK1, IRF-3) signaling partners (13). One possible explanation for the observed differences could be differences in the EBV-status of donors, as STING has been shown to be expressed in EBV-positive B cell lines, but not in EBV-negative cell lines. Both aforementioned studies did not observe IFN-I production from human B cells upon transfection of dsDNA or its synthetic homologs, which could be due to the low transfection efficacy into B cells or point toward an intrinsic defect of the STING signaling pathway in human B cells; however, Dong et al. observed a negative regulatory role of STING signaling in B cells on the JAK1-STAT1 pathway, suggesting a functional role of STING in B cells (12). STING activation has also been shown to upregulate costimulatory molecules, such as CD86, across all B cell subsets, have adjuvant activity following immunization with thymus-dependent antigens, improving antigen-specific antibody responses, and mediate apoptosis both in normal and malignant B cells (14, 15).

Another important nucleic acid sensing pathway in the cytosol is the pathway involving mitochondria antiviral-signaling protein (MAVS, also termed VISA, IPS-1, or Cardif). Several proteins have been suggested to act as cytosolic RNA sensors, including retinoic-inducible gene-I (RIG-I) and melanoma differentiation-associated gene-5 (MDA-5). These sensors can then cause aggregation of MAVS, leading to the activation of IRF-3 and IRF-7, NF-κB and production of IFN-I (16).

Both MAVS and its upstream sensors, RIG-I and MDA-5, have been shown to be expressed in B cells of human and murine origin, and were demonstrated to have a functional sensing pathway. Stimulation of B cells using the synthetic RNA analog poly(I:C) was shown to induce cytokines, predominantly IFN-β and IL-6, and to a lower degree IFN-γ, in a MAVS-dependent manner (13, 17). Additionally, triggering of the RIG-I/MAVS pathway using 5′-ppp-RNA was shown to be an effective adjuvant in influenza vaccination, leading to a long-lasting antibody response of improved specificity (18).

### Pathological Implications of Innate Immune Sensing in B Cells

The identification of a role of innate immune sensing in the cytokine and antibody production in B cells attracted considerable attention in the field of autoimmunity research. Multiple studies have confirmed the involvement of TLR signaling, in particular MyD88-dependent TLRs and endosomal TLR7 and TLR9, in autoreactive B cell activation and germinal center (GC) formation (19), autoantibody production (20, 21), and development of autoantibody-related pathologies such as glomerulonephritis in the context of the human disease systemic lupus erythematosus (SLE) (22, 23) and in models using the lupus-prone mouse strain MRL/lpr (24).

In a mouse model of the IFN-related autoimmune condition Aicardi-Goutières Syndrome (AGS), which is mimicked by a deletion of dsDNA-degrading protein 3′ repair endonuclease 1 (Trex1), B cells were shown to be responsible for the development of glomerulonephritis and greatly contributed to disease-related mortality (25). A different study on lupus demonstrated a negative regulatory effect of STING on JAK1-STAT1 activation and found decreased STING expression in B cells from SLE patients and MRL/lpr lupus-prone mice (12).

MAVS, on the other hand, seems to be involved in regulation of germinal center formation. The formation of spontaneous germinal centers (Spt-GCs), whose dysregulation is associated with SLE and other autoimmune diseases, was shown to be dependent on MAVS and TLR7 expression in mice, and TLR7 ligation could only partially reinstate the Spt-GC development (26). Another study not only confirmed that MAVS in B cells is required for the formation of autoreactive GCs and autoantibody production in lupus-susceptible mice, but additionally linked its expression to the development of proteinuria and glomerulonephritis (27).

Signaling through pattern recognition receptors (PRR) in B cells was also reported to dysregulate processes leading to antibody production. Recent evidence suggests that innate immune activation might directly contribute to detrimental antibody production, as increased TLR7 signaling has been observed to favor differentiation of lupus-associated CD27^−^IgD^−^ B cells into plasma cells excreting autoreactive antibodies, although co-stimulation by IL-21 and IFN- γ along with TLR7 is required to differentiate naïve B cells into these double negative and plasma cells. Furthermore, B cells from SLE patients were found to have increased expression of genes involved in innate RNA sensing, including TLR7, TBK1, and TRIM56, an inducer of STING (28). This is consistent with observations of a prominent IFN-I signature in SLE and other autoimmune diseases, which was shown to further upregulate TLR7 and TLR9 expression, thereby potentially amplifying detrimental autoantibody production (29).

Polyclonal B cell activation and subsequent excessive generation of antibodies, termed hypergammaglobulinemia, is not only a common feature of many autoimmune diseases in humans and in mouse models (30, 31), but is also a hallmark of many chronic infections, including leishmaniasis and Chagas disease (32, 33). As pronounced IFN-I production has been observed in models using *L. donovani* and *T. cruzi* (34, 35), similar mechanisms might be at play to exacerbate B cell activation and cause hypergammaglobulinemia in these diseases.

Not only do autoimmune diseases and chronic inflammatory diseases share many characteristics, including aberrant B cell activation and antibody production, but many pathogens have also been linked to the induction of autoimmune reactions. One of these pathogens is the intracellular protozoan parasite *Trypanosoma cruzi* which induces chronic chagasic cardiomyopathy (CCC) in 30–50% of patients and accompanied by high production of inflammatory cytokines, including IL-1β, IFN-γ, and TNF (36). Different roles for TLRs in *T. cruzi* infection have been proposed—lack of signaling through TLR7 and TLR9 has been found to enhance susceptibility to infection and decrease parasite clearance (37, 38), while TLR2 and TLR4 ligation were shown to modulate the pro-inflammatory response in the cardiac form, and in the anti-inflammatory response in the asymptomatic form of the disease (39); however, little information exists on the contribution of B cell-intrinsic TLRs in this context. Distinct clinical forms of Chagas' disease were found to have different underlying TLR expression and subsequent cytokine production in PBMCs. Elevated levels of TLR2 expression and concomitant production of pro-inflammatory cytokines TNF and IL-12 were found in patients exhibiting cardiac pathologies, while increased TLR8 and IFN-β expression was determined in the digestive forms (40). The frequency of TNF-producing B1 cells in cardiac patients was shown to be higher than in non-infected individuals and was significantly increased upon further exposure to *T. cruzi*-derived protein-enriched fraction (41); however, direct studies on B cells in *T. cruzi* infection are required to elucidate the contribution of B cell TLR signaling to this cytokine production.

Like trypanosomiasis, leishmaniasis is induced by a family of protozoan parasites belonging to the group of Trypanosomatids. Contrasting roles for B cells for different parasite strains have been demonstrated both in disease protection or progression dependent on the model organism and parasite strain [reviewed in (42)]; however, little is known about the contribution of innate immune sensing in B cells in the context of this disease. Using an experimental model of visceral leishmaniasis, our laboratory has previously demonstrated that *Leishmania donovani* amastigotes can induce production of pro-inflammatory cytokines, IFN-I, and IL-10, by engaging endosomal TLR3, TLR7, and TLR9. As *in vitro* exposure of B cells to the parasite also resulted in an IFNAR-dependent upregulation of endosomal TLR mRNA, we proposed a positive regulatory loop of IFN-I on endosomal TLR expression, thereby enhancing the modulatory effect of endosomal TLR signaling on cytokine production and antibody production, which results in hypergammaglobulinemia and disease exacerbation (34, 43). A similar feedback mechanism for IFN-β on TLR7 and TLR3 has been suggested by other groups, and investigation of the source of this IFN-I provides a link between the innate RIG-I/MAVS and TLR signaling pathways (17, 44): Loetsch et al. have found stimulation of the RIG-I/MAVS pathway with synthetic RNA analog to cause upregulation of endosomal TLR expression, namely TLR3 and TLR7, in an interferon-α/β receptor (IFNAR)- and partially MAVS-dependent manner. Thus, IFN-I produced via MAVS-mediated sensing pathways could partially account for the amplification of B cell activation and hypergammaglobulinemia through upregulation of endosomal TLRs; however, the reduction of TLR upregulation in the MAVS-compromised B cells does not fully account for the reduction observed in the *Ifnar*^−/−^ mice. This suggests that there might be an additional source of type-I interferon produced by a MAVS-independent pathway. In fact, DNA derived from *L. donovani* has recently been demonstrated to be able to induce IFN-I production via the cGAS/STING pathway in macrophages (45), and while only the B-1 lineage of B cells has been demonstrated to be able to phagocytose *Leishmania* parasites (46), it is possible that parasite DNA is delivered to the B cell cytosol through yet unidentified pathways to trigger IFN-I production via cGAS/STING.

Finally, while innate immune signaling can thus be subverted by pathogens to exacerbate disease by dysregulating germinal center formation, antibody and cytokine production, targeted engagement of individual sensors, especially of the TLR family, has also been proposed in therapy of various inflammatory and infectious diseases (47); however, the effect of using TLR ligands as adjuvants in therapy can differ widely even in the same model, as demonstrated by a study on established cutaneous leishmaniasis infection caused by *L. (Vianna) panamensis*. TLR9 stimulation using high doses of its ligand CpG was shown to decrease lesion size, drastically reduced parasite burden, and decreased B cell-mediated IFN-γ, while stimulation with low doses of CpG increased IFN-γ production in the same cells (48), highlighting the importance of further studies on innate immune signaling and its effect on adaptive immunity in B cells in order to develop safe and effective treatments for chronic infectious diseases.

The low number of studies on cytosolic nucleic acid sensors in B cells in infectious diseases limits conclusions to be drawn for its relevance in pathological settings at this point in time. The use of a conventional knockout of cGAS in mice was shown to induce dysregulated germinal center and antibody responses and reduced parasite clearance in a non-lethal malaria model; however the effect on GC formation was found to be B cell-extrinsic (49). Similarly, while MAVS^−^ mice infected with the non-pathogenic West Nile Virus-Madagascar (WNV-MAD) strain exhibited increased GC formation, antibody-titers and plasma cell formation, this effect was found to be dependent on MAVS signaling in dendritic cells rather than B cells (50). While both studies chose to focus on the most prominent role of B cells, production of antibodies, neither of them investigated the effect of cytosolic nucleic acid sensing on cytokine production. In B cells isolated from lungs and spleen of *Mycobacterium tuberculosis*-infected mice, a dramatic STING-dependent upregulation of IFN-β mRNA, along with a milder increase in IL-6 and a tendency toward upregulated IL-10 was observed. This marked increase of IFN-β production was also shown to be present in B cells purified from pleural fluid of Mtb patients as compared to healthy donors. Interestingly, a lower amount of IFN-β could also be triggered by poly(I:C), which is a ligand of TLR3 but can also be sensed via the RIG-I/MAVS pathway. Along with another interesting finding that MyD88 signaling suppressed STING-mediated IFN-β expression, this not only provides further evidence of functional cytosolic sensing in B cells in infectious diseases but also draws another connection between cytosolic and TLR sensing (51). Collectively, this implies that, while cytosolic sensing in B cells might have a limited direct effect on GC formation and antibody response, it can have substantial effect on modulating cytokine production, and, through interconnections between the different innate immune sensing pathways, potentially mediated through IFN-I, a central player in many of these pathways, indirectly mediate B cell responses.

Despite studies demonstrating the functionality of intracellular innate immune sensing pathways in B cells, the question still remains as to how these mostly non-phagocytic immune cells are able to recognize pathogen-derived nucleic acids in their cytosol. One possibility has been demonstrated in the case of *Listeria monocytogenes*, which can induce nucleotide sensing in non-phagocytic cells by secretion of bacteria-derived nucleic acids (52, 53). As many chronic infections are characterized by increased apoptosis and tissue disruption, as the case during hepatosplenomegaly in visceral leishmaniasis or cardiac damage in Chagas' disease (54, 55), it is also possible that this may promote release of host nucleic acids into the tissue environment (56), which could in turn induce innate immune sensors in surrounding cells, including B cells. Nevertheless, further investigations are warranted to identify the mechanisms underlying activation of intracellular sensors in B cells by pathogens.

**Figure 1:**
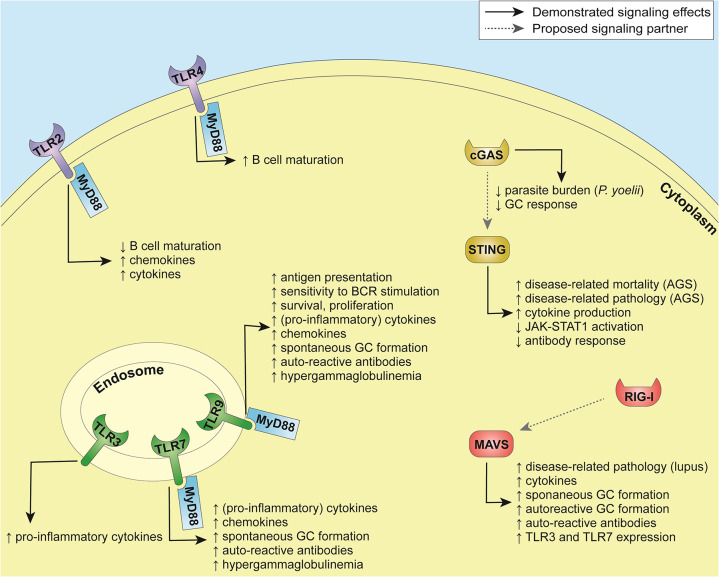
Summary of pathways involved in innate immune sensing in B cells.

Functional outcomes of PRR triggering in B cells are summarized in Table 1 and Figure 1.

**Table 1:** Innate immune sensing in B cells.

**PRR/adaptor**	**Function**	**Model/agonist**	**Organism**
TLR2	↓ B cell maturation (5)	Pam3Cys	Mouse
	↑ Cytokine and chemokine production (8)	Pam3CSK	Human
TLR3	↑ Pro-inflammatory cytokines (34)	*Leishmania donovani*	Mouse
TLR4	↑ B cell maturation (4, 5)	LPS	Mouse
TLR7	↑ Cytokine and chemokine production (8)	Imiquimod	Human
	↑ Spontaneous GC formation (19, 26)	Imiquimod	Mouse
	↑ Autoantibody production (20)	Lupus-prone mice	
	↑ Pro-inflammatory cytokines, ↑ hypergammaglobulinemia (34)	*Leishmania donovani*	
TLR9	↑ Proliferation, ↑ survival, ↑ costimulatory molecule expression, ↑ antigen presentation (6), ↑ sensitivity to BCR stimulation (7), ↑ cytokine and chemokine production (8)	CpG (6, 7), GpG-ODN2006 (8)	Human
	↓ Spontaneous GC formation (19)	TLR9 knockout	Mouse
	↑ Autoantibody production (20)	Lupus-prone mice	
	↑ Pro-inflammatory cytokines, ↑ hypergammaglobulinemia (34)	*Leishmania donovani*	
	At high CpG doses (> 1 μM): ↓ lesion size, ↓ parasite burden, ↓ IFN-γ at low CpG doses (>40 nM) ↑ IFN-γ	CpG treatment in *Leishmania (Vianna) panamensis*	
cGAS	↓ Parasite burden, ↓ GC response (49)	*Plasmodium yoelii*	Mouse
STING	↑ Disease-related mortality, ↑ glomerulonephritis (25)	Aicardi-Goutières Syndrome	Mouse
	↓ JAK-STAT1 activation, ↓ antibody response (12)	Systemic lupus erythematosus (SLE)	Mouse/human
	↑ Cytokine production (51)	*Mycobacterium tuberculosis*	
MAVS	↑ Spontaneous GC formation (26), ↑ autoreactive GC formation, ↑ autoantibody production, ↑ disease-related pathology (27)	Lupus-prone mice	Mouse
	↑ TLR3 and TLR7 expression (17), ↑ cytokine production (51)	Poly(I:C)	

## Innate Immune Sensing in T Cells

### Expression and Functions of Innate Immune Sensors in T Cells

T lymphocytes have also been reported to express several PRRs; however, the downstream effect of PRR activation varies depending on T cell population, activation status, ligand, and/or environment.

Most of the available literature on PRR expression in T lymphocytes investigates the role of TLRs in T cell differentiation and effector function. Murine and human T cells were shown to express mRNA and protein for most of the TLRs (57–64); however, their expression intensity depends on T cell subsets and activation status. Interestingly, TLR expression seems to be regulated by TCR-dependent activation; indeed, antigen-experienced T cells express higher TLR levels than naïve cells (58, 59, 65). During priming, TLR activation appears to function as a sort of costimulatory signal enhancing effector function, proliferation, cell survival, and cytokine production in murine and human CD4 T cells (61, 64, 66, 67). For example, signaling through TLR9 can induce NF-κB activation in CD4 T cells via the adaptor molecule MyD88, leading to the upregulation of anti-apoptotic molecules and increasing cell survival (58). Similar anti-apoptotic functions were ascribed to TLR2 in CD8 T cells (68). Moreover, signaling throughTLR9- MyD88 also promotes CD4 T cells proliferation by activating a PI3K/Akt-dependent pathway (67).

Expression of costimulatory molecules and cell trafficking are also promoted by TLR stimulation. For instance, CpG ODN (TLR9 agonist) induces expression of OX-40 and CD40L on CD4 T cells during priming; while treatment with LPS increases adhesion capacity and inhibits chemotaxis of human and murine T cells (69, 70).

The importance of intrinsic MyD88-dependent signals in promoting CD8 and CD4 T cell survival and initial proliferation was also demonstrated in *in vivo* studies in various models of infection, including *Toxoplasma gondii* and Lymphocytic choriomeningitis virus (LCMV) infections (71, 72).

Moreover, MyD88 and TLR signaling appears to be essential for the differentiation of Th17. cells. The vital role of MyD88 was shown in experimental models of colitis and experimental autoimmune encephalitis (EAE). In the colitis model, *Myd88*^−/−^ CD4 T cells showed reduced survival, failed to induce severe disease, and poorly differentiated into Th17 cells (60, 73). Later studies in the EAE model suggested TLR2 and TLR4 signaling as being crucial for the differentiation of Th17 cells (64, 74, 75). Indeed, TLR2 activation in CD4 T cells seems to synergize with IL-23 to induce Th17 cells; additionally, TLR2-deficient CD4 T cells fail to induce EAE and to differentiate into IL-17 or IFN-γ-producing cells in adoptively transferred mice.

Taken together, the literature suggests that TLR signaling plays an important role in providing cell survival and proliferative signals during T cell priming and in enhancing effector functions and cell differentiation.

Some cytosolic nucleic acid sensors were also reported to be expressed in T cells. For instance STING expression was detected in human and murine T cells (76–78); RIG-I is expressed in human peripheral T lymphocytes (79); LGP2 is present in murine CD8 T cells (80); and the immune sensor NLRC3 was observed in murine CD4 T cells (81). With exception of LGP2, all other pathways appear to impair T cell proliferation, function, or survival. Hence, their role in T cells will be discussed in the next section. LGP2 is a member of the RIG-I-like receptors family of cytosolic RNA helicases that includes RIG-I and MDA5. Unlike RIG-I and MDA5, which are known to initiate the activation of IRF-3 and NF-κB to induce expression of IFN-I, LGP2 can function as a negative regulator of RLR signaling inhibiting TLR-independent sensing of viral replication (82) and RIG-I multimerization (83), or compete with MAVS to suppress innate immune signaling (84). A positive function for LGP2 as a cofactor for RLR signaling of RIG-I and MAVS-mediated antiviral responses has also been described, but the mechanism is yet unknown (85, 86). In CD8 T cells, LGP2 promotes cell fitness and survival by controlling sensitivity to death-receptor signaling during acute West Nile virus and LCMV infections. Indeed, LPG2-deficient CD8 T cells display enhanced activity of caspase 8, 3, and 7 and enhanced expression of death receptors TNFR-I, TRAILR2 (or DR5), and CD95 (or Fas receptor) (80).

### Immunosuppressive Effects of Innate Immune Sensing in T Cells

Despite the strong evidence that T cell-intrinsic PRR activation complements TCR and costimulatory signals to improve T cell responses during priming, a few studies have reported an inhibitory role for innate immune sensing in T cells.

Signaling via TLR2, for instance, was shown to inhibit T cell chemotaxis through upregulation of the transcription factor SOCS3 (suppressor of cytokine signaling 3) (87). An additional study reported that TLR2 was also involved in downregulating the transcription factors T-bet and NF-κB (88). Both studies used Heat shock protein 60 (HSP60), arguably a DAMP, to stimulate T cells. In contrast, in CD25+ CD4+ regulatory T cells (Tregs) exposed to HSP60 upon activation with anti-CD3, TLR2 was required to enhance their immunosuppressive effects via activation of PCK, PI-3 kinase, and p38 (89). Stimulation of Tregs with pathogen-derived TLR2 ligands induced proliferation and promoted survival (65, 89–91); however, whether this enhances (89, 91) or curbs (65, 90) their inhibitory function is still controversial.

While TLR4 activation is essential to drive Th17 responses (75), its effects on Th1 cells are rather inhibitory in a spontaneous model of colitis (92) and in human T cells exposed to LPS (70), where signaling via TLR4 inhibited cell migration. Inhibitory effects were also ascribed to the TLR7 activation pathway in CD4 T cells. In a model of EAE, triggering of TLR7 suppressed Th17 cell differentiation, which resulted in reduced disease severity (93). This effect was mediated by downregulation of STAT3 and induction of SOCS3 and SOCS5 (93). Furthermore, in human T cells purified from the blood of HIV^+^ individuals, TLR7 stimulation promoted the activation of an NFATc2-dependent anergic gene-expression program, which led to cell unresponsiveness (62). Work from our laboratory has also revealed an inhibitory function for TLR7 activation in Th1 cells in *L. donovani* infected mice. During the chronic stage of disease, Th1 cells increasingly upregulated TLR7 expression and sensed DAMPs derived from inflammatory tissue disruption (59). Engagement of TLR7 on those cells resulted in the activation of the transcription factor interferon regulatory factor 5 (IRF-5), which induced the transcriptional activation of death receptor 5 (DR5 or TRAILR2) and caspase 8, thereby promoting cell death (59).

TLRs are not the only innate immune sensors capable of inhibiting T cell functions. The STING pathway was recently shown to be active in T cells as well. Stimulation with STING agonists not only promoted IFN-I production and interferon stimulated genes' expression, but it also led to the down-regulation of anti-apoptotic and the upregulation of pro-apoptotic genes (78). Interestingly, T cells exhibit an intensified STING pathway, which results in a different gene expression profile to innate myeloid cells and leads to cell death (94). Moreover, activation of STING was shown to have an antiproliferative effect in human and murine CD4 T cells. This antiproliferative capacity requires STING relocalization to the Golgi apparatus (76).

The RIG-I pathway seems to also have inhibitory effects in human T cells. Zhang et al. report a positive correlation between RIG-I expression in peripheral T cells and T lymphocyte counts in patients affected by dermatomyositis. Interestingly, RIG-I induced apoptosis in these cells and inhibited their proliferative capacity (79).

Another molecule involved in innate immune sensing pathways that was recently shown to reduce T cell effector functions is NLRC3 (95). NLRC3 belongs to the group of non-inflammasome-forming NLRs (NOD-like receptors), together with NOD-1, NOD2, among others. NLRC3 is a known negative regulator of innate immunity and inflammatory responses (95, 96). This molecule is highly expressed in T cells, where it seems to fine-tune CD4 T cell activation by attenuating IFN-γ and TNF expression, decreasing proliferation of Th1 and Th17 cells, and affecting cell metabolism by reducing glycolysis and oxidative phosphorylation (81).

**Figure 2:**
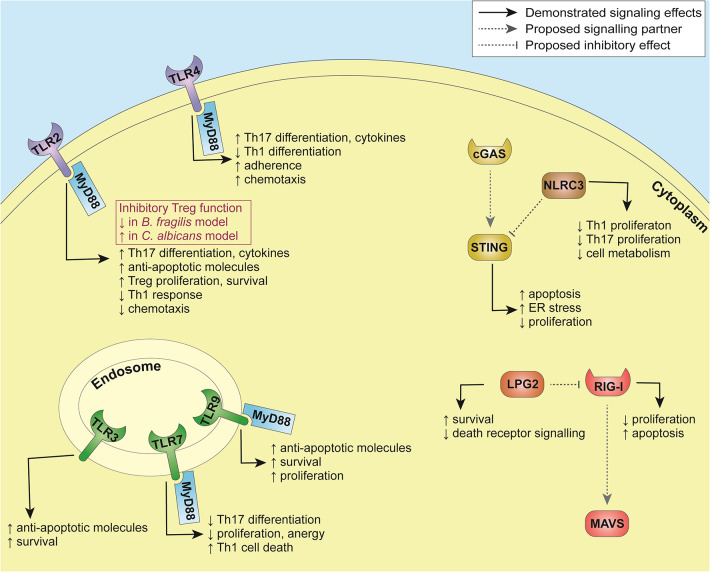
Summary of pathways involved in innate immune sensing in T cells.

It was also demonstrated that T cells are capable of sensing nucleic acids via pathways distinct of those identified so far in the innate immune system. Interestingly, higher-order structure of the nucleic acids was required for their internalization by T cells; indeed, self-DNA released from dead cells and complexes with antimicrobial peptides or histones induced costimulatory responses upon recognition by yet unidentified sensor(s), promoting the differentiation into Th2 cells (97). Downstream effects of innate immune sensing in T cells are summarized in Table 2 and Figure 2.

**Table 2:** Innate immune sensing in T cells.

**PRR/adaptor**	**Function**	**Model/agonist**	**Organism**
MyD88	↑ Disease susceptibility (71)	*Toxoplasma gondii*	Mouse
	↑ Clonal expansion, ↑ survival (72)	Lymphocytic choriomeningitis virus (LCMV)	
	↑ Survival, ↑ disease induction, ↑ Th1 and Th17 differentiation (60, 73) ↑ Expression of anti-apoptotic molecules (73)	Colitis	
TLR2	↑ Anti-apoptotic molecules, ↑ Survival, ↑ proliferation (65, 68)	Pam(3)CysSK(4)	Mouse
	↑ Th17 differentiation, ↑ Th17 cytokine production (64)	Experimental autoimmune encephalomyelitis (EAE)	
	↑ Tregs proliferation, ↑ survival (65, 89–91) ↑ Inhibitory Treg function (89, 91) ↓ Inhibitory Treg function (65, 90)	Pam3CysSK4 (65), HSP90 (90), *Candida albicans* (91) Bacteroides fragilis (92)	
	↓ Chemotaxis (87), ↓ Th1 responses (88, 89)	HSP60	Human/mouse
TLR3	↑ Anti-apoptotic molecules, ↑ Survival (58)	Ligation with poly(I:C)	Mouse
TLR4/MyD88	↑ Adherence, ↑ chemotaxis (70)	Ligation with LPS	Human
TLR4	↑ Th17 differentiation, ↑ Th17 cytokine production (75)	EAE	Mouse
	↓ Th1 responses (92)	Colitis	
TLR7	↑ Th1 cell death (59)	*Leishmania donovani*	Mouse
	↓ Th17 differentiation (93)	EAE	
	↓ Proliferation, anergy (62)	HIV-1	Human
TLR9/MyD88	↑ Anti-apoptotic molecules, ↑ Survival (58), ↑ proliferation (67)	Ligation with CpG	Mouse
RIG-I	↓ Proliferation, ↑ apoptosis (79)	Dermatomyositis	Human
LPG2	↑ Survival, ↓ death receptor signaling (80)	RNA viruses	Mouse
STING	↓ Proliferation (76)	Activating STING mutations	Human/mouse
	↑ ER stress, ↑ apoptosis (78, 94)	DMXAA (78), CMA (95)	Mouse
NLRC3	↓ Th1 proliferation, ↓ Th17 proliferation, ↓ Cell metabolism (81)	LCMV, EAE	Mouse
Unknown nucleic acid sensor	↑ Co-stimulatory responses, ↑ Th2 differentiation (97)	Ligation with TLR ligands, synthetic NA analogs, self-DNA from dead cells	Mouse

It is interesting to observe that innate sensing by T cells during the priming phase leads mainly to a positive outcome: it promotes cell survival, enhances effector function, and helps differentiation. In contrast, during chronic infections or in a chronic inflammatory environment, PRR activation in T cells results in cell death and anergy, and limits the cells' proliferative capacity. It is yet unclear why PRR signaling mediates such disparate functions depending on the cell differentiation stage and the inflammatory environment. Further studies are definitely warranted to better define the level of expression, signaling pathways and downstream targets of PRRs in various T cell populations. Also, very little information is currently available on innate immune sensing by T cells through PRRs that are upregulated during chronic infection and the importance these may have in helping pathogen persistence and inhibiting protective T cell responses. Furthermore, the nature of the ligands responsible for triggering those responses in T cells, and the stimuli required for promoting PRR expression during chronic infections have also not yet been identified. We have demonstrated that DAMPs could trigger TLR7 and induce cell death in T cells isolated from *L. donovani*-infected mice during chronic infection (59). Inflammatory tissue damage is a common characteristic of persistent infections and release of several DAMPs through this process is inevitable. It is thus possible that other PRR, such as STING and RIG-I, are also activated by DAMPS derived from tissue damage during chronic visceral leishmaniasis. While curbing of pro-inflammatory T cell responses in a chronic inflammatory environment could represent a protective mechanism to prevent tissue disruption, it may also favor pathogen persistence. Indeed, in our model, disruption of the TLR7 signaling pathway in T cells resulted in stronger Th1 responses and a lower parasite burden (59). Hence, it would be interesting to investigate the role of PRRs in T cells during other parasitic infections to identify pathways that could possibly be exploited for therapeutic purposes.

## Concluding Remarks

An important body of literature has now demonstrated that PRRs are expressed and functional in cells of the adaptive immune system. In these cells, PRR activation can support signaling pathways that are beneficial to host immunity or, on the contrary, promote adverse effects that favor pathogen persistence. In light of this information, it is thus important to consider the effect of TLR agonists on T and B cells, and not only on myeloid cells, when developing new vaccination strategies, particularly for therapeutic purposes. Indeed, endosomal TLR agonists' administration during chronic stages of infection may lead to CD4 T cell death and/or exacerbate hypergammaglobulinemia. The same caveat may also be valid for immunotherapeutic interventions involving TLR agonists, which could have disease-exacerbating consequences if the wrong cells are involuntarily targeted.

Further investigations are required to better define signaling pathways and downstream targets of PRRs in T and B cells in the context of chronic infections, since these pathways could be exploited for novel therapies.

## Author Contributions

TS and SS made substantial, direct, and intellectual contribution to the work and wrote the manuscript.

## Conflict of Interest

The authors declare that the research was conducted in the absence of any commercial or financial relationships that could be construed as a potential conflict of interest.
